# A Perspective on the Flame Spray Synthesis of Photocatalyst Nanoparticles

**DOI:** 10.3390/ma6083194

**Published:** 2013-07-31

**Authors:** Wey Yang Teoh

**Affiliations:** Clean Energy and Nanotechnology (CLEAN) Laboratory, School of Energy and Environment, City University of Hong Kong, Tat Chee Avenue, Kowloon, Hong Kong; E-Mail: wyteoh@cityu.edu.hk; Tel.: +852-3442-4627; Fax: +852-3442-0688.

**Keywords:** flame synthesis, photocatalysis, semiconductor, nanoparticle, mixed oxides

## Abstract

The synthesis of functional nanoparticles via one-step flame spray pyrolysis (FSP), especially those of catalytic nature, has attracted the interests of scientists and engineers, as well as industries. The rapid and high temperature continuous synthesis yields nanoparticles with intrinsic features of active catalysts, that is, high surface area and surface energetics. For these reasons, FSP finds applications in various thermally inducible catalytic reactions. However, the design and synthesis of photocatalysts by FSP requires a knowledge set which is different from that established for thermal catalysts. Unknown to many, this has resulted in frustrations to those entering the field unprepared, especially since FSP appears to be an elegant tool in synthesising oxide nanoparticles of any elemental construct. From simple oxide to doped-oxide, and mixed metal oxide to the* in situ* deposition of noble metals, this Perspective gives an overview on the development of photocatalysts made by FSP in the last decade that led to a better understanding of the design criteria. Various challenges and opportunities are also highlighted, especially those beyond simple metal oxides, which perhaps contain the greatest potential for the exploitation of photocatalysts design by FSP.

## 1. Flame Spray Synthesis of Nanoparticles

Flame aerosol synthesis is an established manufacturing technique for producing commodity nanoparticles at the industrial scale. Its modern history stretches as far back as the 1940s, when Degussa (now Evonik) patented and manufactured Aerosil^®^ fumed SiO_2_ from the flame hydrolysis of SiCl_4_ vapour [1]. Today, a variety of particles are produced using the flame technique, in descending order of production mass: carbon blacks, pigmentary TiO_2_, ZnO, fumed SiO_2_ and optical fibers with an estimated annual production on the order of million metric tons [2]. Importantly, TiO_2_ nanoparticles manufactured by Evonik under the brand name of P25 has survived the test of time as the gold standard photocatalyst. Similarly to the original Aerosil process, these photocatalytic particles are manufactured by the hydrolysis of TiCl_4_ vapour in an oxy-hydrogen flame. Although the target market was geared towards commodity pigments, since 1980s, P25 TiO_2_ nanoparticles have been routinely applied to various photomediated processes, namely, purification of air and water [3,4], organic syntheses [5], water splitting [6] and dye-sensitised solar cells [7]. The high intrinsic activity of this reference photocatalyst has proven difficult to be overtaken by many other designer TiO_2_ particles. So it is intriguing that the origin of high activity of P25 is still a subject of intense investigation [8,9,10].

As a modern variation of flame synthesis, many research groups [11,12,13,14], including us [15], have been actively developing the flame spray pyrolysis (FSP) for a plethora of applications. Unlike the conventional vapour-fed flame synthesis (VFS) [13] used to manufacture P25 TiO_2_, the FSP features the direct combustion of highly exothermic liquid precursor sprays. Whereas metal precursors are thermally vaporised prior to feeding into the VFS flame, here, the FSP makes use of the high flame temperature to vaporise the liquid precursor at the upstream of the flame itself. There are many advantages of pursuing the latter path, that is, switching from vapour to liquid precursors. They include circumventing the challenges of handling toxic metal carbonyls or corrosive metal halides vapours during the precombustion process, and/or by products such as HCl from the hydrolysis of metal halides in the post-combustion process. More importantly, a much wider choice of metal precursors can be made available in FSP since it is less restricted by the requirement of volatile precursors necessary for VFS. In fact, the current selection of metal precursors for FSP is so wide the reported metal precursors cover almost half of all the elements in the periodic table [15].

**Figure 1:**
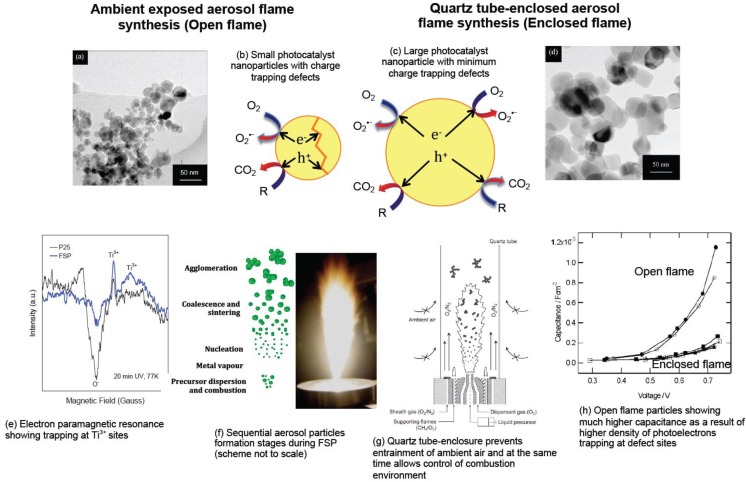
Transmission electron micrograph of TiO_2_ nanoparticles prepared in an (**a**) open ambient FSP; and (**d**) quartz tube-enclosed FSP, adapted with permission from [45]; Schematic illustration of (**b**) small open flame-synthesised photocatalyst with charge trapping defects; and (**c**) large enclosed flame-synthesised photocatalyst with minimum charge trapping defects; (**e**) Compared to commercial P25, as-prepared open flame FSP TiO_2_ particles show the presence of more reductive sites,* i.e.*, Ti^4+^ + e^−^ → Ti^3+^, as measured by electron paramagnetic resonance (EPR) under UV exposure at 77 K; (**f**) Schematic of the process of aerosol nanoparticles formation by the gas-to-particle route (not to scale) and a photograph of the actual aerosol flame; (**g**) Schematic diagram of the quartz-tube enclosed FSP preventing the entrainment of ambient air thereby prolonging the high temperature region in the aerosol flame, adapted with permission from [50]; (**h**) Capacitance measurements of FSP TiO_2_ synthesised in an open flame shows more charge traps (hence higher capacitance), compared to those synthesised in a quartz tube-enclosed flame, adapted with permission from [49].

For formation of homogeneous particles, as that in VFS, highly exothermic liquid precursors are essential during FSP to ensure that the metal precursors are completely vaporised within the flame. Once they exist as metal/metallorganic vapours, the desired metal oxide particles can be formed via the vapour-to-particle route, a process similar to a VFS (Figure 1f, and to be explained below) [1,16]. Direct utilisation of the lowest cost precursors such as nitrates and acetates are unsuitable for FSP due to their low combustion enthalpies as they may not even vaporise completely in the flame. Nevertheless, formulations exist requiring only basic reflux and distillation, to convert them into highly exothermic organometallics [16,17,18]. Metal alkoxides, 2-ethylhexanoates, acetylacetonates are generally preferred, since they also have low boiling points relative to that of the solvent medium [15]. This allows the vaporisation of metal precursors prior to the full vaporisation of the organic solvent at the early stage of the flame combustion [19]. Once metal vapour is formed, the remaining downstream process includes the sequential nucleation as metal or metal oxides, sintering and coalescence, aggregation and agglomeration (Figure 1f) [20,21]. In the cases of non-volatile precursors with low combustion enthalpy (sometimes endothermic, e.g., metal nitrates), this may lead to the formation of solid precursor residue from each spray droplet, much like a spray drying process (*i.e.*, droplet-to-particle route). This would lead to large and overall inhomogeneous particles.

One can see that in FSP, the amount of metal elements can be effectively conserved within the flame and further condensed as the product particles. In other words, the stoichiometry of the resultant particles is fully reflected as originally designed in the liquid precursors. This feature, coupled with the wider availability of liquid precursors, has to date facilitated the rapid expansion of flame syntheses of complex functional nanoparticles consisting of two or more metal elements. They range from doped metal oxides, (Al-doped CeO_2_ [22], Cu-doped Ce*_x_*Zr_1–*x*_O_2_ [23,24,25], Sn-doped ZnO) [26]; decoration of metal deposits on oxide supports (Ag/TiO_2_ [27], Pt/Al_2_O_3_ [28], Rh/Al_2_O_3_ [29], Pd/Fe_2_O_3_) [30]; surface encapsulation (SiO_2_/ZnO [31], Al_2_O_3_/Ce_0.7_Zr_0.3_O_2_ [32], SiO_2_/Fe_2_O_3_ [33,34], TiO_2_/CeO_2_ [35], C/Pt [36]); mixed metal oxides (LiFe_5_PO_8_ [37], LaCoO_3_ [38], SrTiO_3_); and heterojunctions (BaCO_3_/Al_2_O_3_) [39]; to solid solutions (Ce*_x_*Zr_1–*x*_O_2_) [40]. Here, the flexibility of designing particles in a mix-and-match and scalable manner, creates an emerging market for flame-made materials, as it diversifies beyond commodity-focused simple oxides. Niche functional nanoparticles with high market values, for instance, Ta_2_O_5_/SiO_2_ dental fillers are lucrative and commercially very attractive. Despite being only at the dawn of a very exciting frontier in high-tech applications, heterogeneous thermal catalysis is currently leading in terms of capitalising the versatility of the FSP in synthesising various types of oxide catalysts [41]. By comparison, the extent of photocatalysts exploration by FSP has been somewhat limited. This arises from the different sets of design requirements for high efficiency photocatalysts, where the most frequently encountered issue in flame-made photocatalysts is the high defects content which acts as charge trapping or recombination centres.

Whilst the extremely short residence time of flame particles formation (typically in the range of milliseconds, that is, from the liquid precursor spray right up to agglomerated particles leaving the flame), was particularly advantageous in fabricating nanoscale particles, the time spent by the aerosol particles at high temperature flame zone was simply too short for sufficient defects healing to take place [42,43]. This is in contrast to the syntheses by solid-state sintering, where materials are typically subjected to prolonged high temperature exposure (>1273 K, >6 h) to yield oxide crystals with minimum defects as necessary for applications in superconductors and solar water splitting [44]. That is not to say the flame-made particles are amorphous, in fact, these as-prepared particles are often highly crystallined single crystals when observed under high-resolution transmission electron microscopy (HRTEM) and X-ray diffraction (XRD) [45]. Rather, these particles are populated by high-density atomic defects, which can only be observed by certain molecular probes or reactions, or other specialised spectroscopic techniques.

It is easy to understand that the surface defects are advantageous for thermal catalytic reactions since these are the surface energetic sites. In some cases, foreign dopants are deliberately introduced during flame synthesis to induce additional surface defects to the crystals in order to improve catalytic efficiencies [22]. As photocatalysts however, these otherwise active sites become the electron-hole pair charge trapping (deep traps) and recombination centres. In other words, the detrimental effect of charge recombination overwhelms the benefits of surface energetics. The design of high-energy reactive facets with minimal charge recombination centres would be an ideal photocatalyst in this case, but this has so far only been demonstrated by wet preparation techniques [46]. Hereby in this Perspective, we revisit some case studies in the development of photocatalysts by FSP, highlighting the progress as well as the scientific and technical challenges in bridging the FSP and photocatalysis.

## 2. Simple Metal Oxide Photocatalysts—Starting from the Basics

In 2005, we reported our first attempt to synthesise TiO_2_ photocatalysts by FSP and showed improvement over P25 TiO_2_ in the photocatalytic mineralisation of sucrose [47]. As we later found out, the FSP-madeTiO_2_ consistently outperformed P25 (despite both having similar anatase:rutile ratio, the two most common polymorphic phases of TiO_2_) in the degradation of saccharides, that is, sucrose, glucose and fructose [48]. The trapping of photoelectrons at the surface defect sites of FSP TiO_2_, as confirmed by electroparamagnetic resonance (Figure 1e), was thought to be beneficial in the direct charge transfer to the saccharides that enhanced degradation. However, when the same FSP TiO_2_ was assessed for the photocatalytic mineralisation of phenol and methanol, it showed inferior activity compared to P25 [48]. This was traced to the fact that these compounds are both hydroxyl radical scavengers, which the FSP TiO_2_ showed approximately 10% less efficiency in generating free hydroxyl radicals compared to P25 [48]. Such example where a photocatalyst shows high efficiency in the degradation of a single class compound but less efficiency in another is not unusual. In fact, it is a general phenomenon since every photocatalyst has its own distinct physicochemical characteristic that favours certain degradation pathways. Given the infinitely large number of organic substrates and their degradation pathways, it should be stressed here that there is not one photocatalyst that fits all.

While the electron-trapping surface defects may appear to favour the degradation of saccharides, the high defects content in conventionally-prepared FSP photocatalysts slow down the bulk electron diffusion since the photoelectrons are shallowly and deeply trapped at these sites [49]. The limitation can be overcome by synthesising the TiO_2_ in a quartz tube-enclosed FSP (Figure 1g) [50]. By doing so, the free entrainment of ambient air can be prevented, and hence the flame residence time at high temperature regions can be prolonged for defects healing. As a result, the capacitance, or the amount of charge trapping defects, was reduced by an order of magnitude (Figure 1h), but at the expense of larger crystallite size since the longer residence time at high temperature zone resulted in severe sintering of the aerosols [49,51]. Typically, the product particle size is increased by a few-fold compared to the conventional open, non-enclosed flame (Figure 1a–d).

In TiO_2_ photocatalysis, the hypothesis of synergetic photocatalytic effect surrounding mixed TiO_2_ anatase-rutile polymorphs is a subject of fundamental interest. Optimal mixture of anatase and rutile exists where it is photocatalytically more active than their respective pure states [52,53]. This prompted us to design TiO_2_ photocatalysts with controllable anatase-rutile content by FSP. By regulating the ambient oxygen partial pressures of the aerosol flame within the quartz tube enclosure, it is possible to tune from almost pure anatase to rutile by decreasing the combustion oxygen partial pressures [50]. Perhaps quite uniquely compared to other approaches of TiO_2_ polymorphic engineering, the enclosed flame synthesis approach resulted in similar crystallite sizes independent of the polymorph content. This could not be easily achieved by conventional thermal annealing of anatase TiO_2_, where the rutile content increases as a function of temperature above 400 °C with corresponding particles sintering [54]. By synthesising an entire range of anatase-rutile concentration independent of size by enclosed FSP, we reported a wide range of anatase-rutile concentration window (13%–79% anatase, the remaining as rutile) for which the synergetic hydrogen evolution can be observed [45]. This was attributed to the favourable charge separation across anatase-rutile interface, but such an effect was only possible when the two phases exist in intimate interparticle contact. Physical mixture by grinding of the equivalent amount of preformed anatase and rutile could not reproduce the synergetic effect since the two phases are only contacted at inter-aggregate level [45].

**Figure 2:**
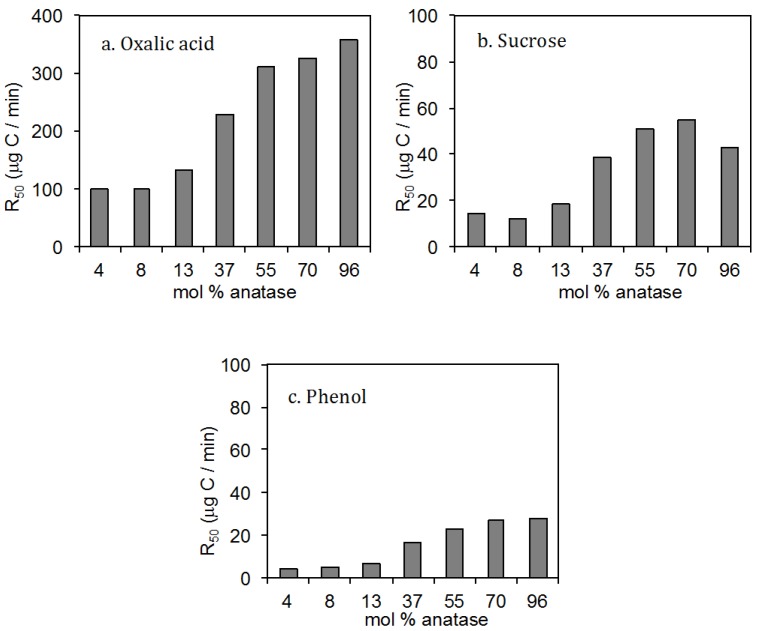
Half-life mineralisation rates of (**a**) oxalic acid; (**b**) sucrose and (**c**) phenol over TiO_2_ nanoparticles of different anatase content (Rutile (mol %) = 100% minus Anatase) prepared in a quartz tube-enclosed FSP. Catalyst loadings 0.2 g/L; Initial carbon loading 2000 µg; Initial pH 3.0; Light source: 16 W Blacklight blue fluorescence. Data courtesy of Rose Amal and coworkers [55].

Extending the studies further, we investigated the polymorphic effect of FSP TiO_2_ nanocrystals on the photocatalytic degradation of different organic compounds. As shown in Figure 2b, a synergetic photocatalytic mineralisation of sucrose can be observed in the range of 37%–70% anatase. In this instance, the enhanced charge separation at the anatase-rutile interface was thought to be beneficial in direct interfacial charge transfer to sucrose, as consistent with earlier discussion on the mineralisation of saccharides by FSP TiO_2_. For other classes of compounds, namely oxalic acid (Figure 2a) and phenol (Figure 2c), the mineralisation rates appear to be favoured by high anatase content. Many polymorphic studies also reported favourable activity over anatase TiO_2_, tracing it to the higher yield of hydroxyl radicals [54]. These results reiterate our earlier message that the efficacy of a photocatalyst is highly specific to the degradation mechanistic pathways of the organic substrates.

Moving away from TiO_2_, ZnO is one of the leading alternative photocatalysts, which has similar conduction and valence band edges as TiO_2_. Its syntheses by FSP have been relatively wellexplored [26,56]. Height* et al.* [57] showed comparable reaction kinetics between FSP-made ZnO and P25 TiO_2_ in the photocatalytic degradation of methylene blue dye. However, the biggest concern of ZnO is its chemical stability,* i.e.*, materials corrosion, both in the dark as well as under photoexcitation. Under intrinsic excitation, the photo-generated holes weaken the Zn–O bonds, resulting in soluble Zn^2+^ ions and O_2_ [58]. For this reason, it is essential to monitor soluble Zn concentration during photocatalysis, especially in the presence of complexing agents which may further aggravate the effects of dissolution [59,60].

WO_3_ is an interesting visible light-driven, mid-bandgap photocatalyst, for which the conduction band edge (+0.5 V* vs.* NHE) is more positive than the redox of superoxide radical formation: *E*(O_2_/O_2_˙^ −^) = −0.33 V *vs.* NHE [61]. In other words, the standard practice of air or oxygen purging during photocatalytic assessment will be ineffective for WO_3_, at least when used without co-catalyst. To overcome this, co-catalysts such as Pt deposits are often introduced to promote alternative multiple electron reduction of oxygen: O_2_ + 2H^+ ^+ 2e^−^ → H_2_O_2_ (+0.695 V* vs.* NHE), and O_2_ + 4H^+^ + 4e^−^ → 2H_2_O (+1.23 V* vs.* NHE). In terms of FSP synthesis, a suitable commercial tungsten precursor with high enough concentration for high throughput synthesis of pure WO_3_ has been a major hurdle in the past, as only 10 vol % tungsten 2-ethylhexanoate in hexane is available commercially. In recent years, the high concentration formulation of tungsten benzoate through the spontaneous reaction of tungsten hexachloride with benzoic acid has substituted for such needs [62]. Despite yielding highly crystalline WO_3_ nanocrystals from FSP using this precursor, no published data on its photocatalytic characteristics is yet available, be it in its pristine form or that loaded with co-catalysts. In any case, it is essential to compare both states to obtain fundamentals information on its photo-physicochemical properties as well as its ability to catalyse multiple electrons reduction of O_2_ when decorated with co-catalysts.

As general good practice, care should be taken when assessing the efficiencies of photocatalysts with regard to the choice of organic substrates and the light source. We take the opportunity to echo the message by Professor Bunsho Ohtani [63,64], which has been wellreceived within the photocatalysis community, that the effects of organics (and more prominently dyes) degradation by photosensitisation-degradation and photocatalytic oxidation should be distinguished. In the former, dyes absorb suitable photons resulting in the excitation of ground state electrons from HOMO to LUMO. The LUMO electrons then inject into the conduction band of the photocatalyst. In the case if the electrons are not regenerated at HOMO, the dye will degrade, even without photogenerated charges from the photocatalyst. In other words, the degradation of dyes cannot fully reflect the photocatalytic efficiencies of the photocatalysts.

**Figure 3:**
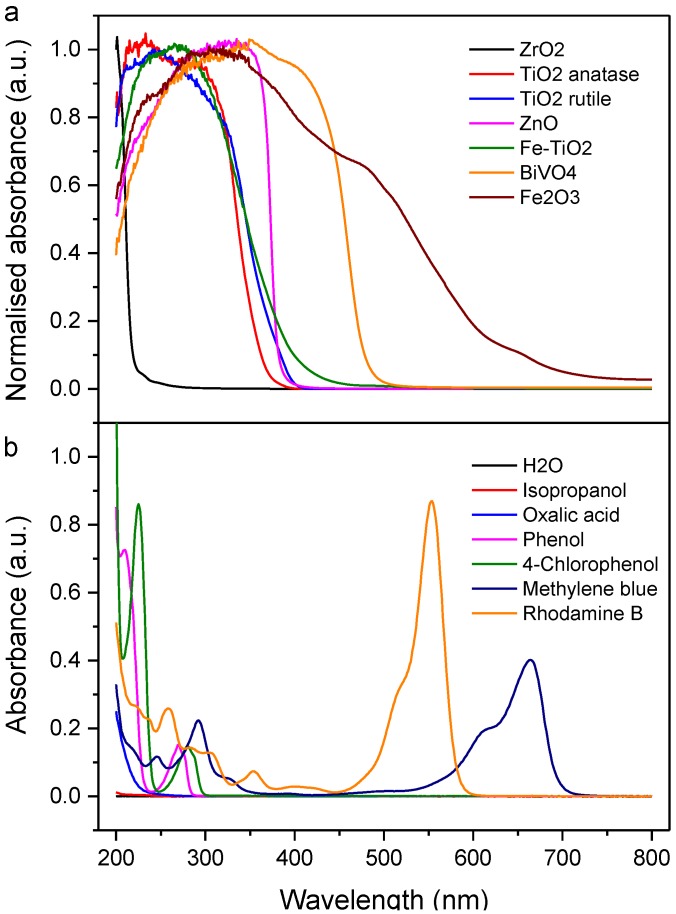
(**a**) Normalised Kubelka-Munk absorbance of various photocatalysts prepared by FSP as measured in diffuse-reflectance mode in an integrating sphere; and (**b**) absorbance of a range of commonly employed substrates (0.1 mM) used in the assessment of photocatalysts.

Figure 3 shows the optical absorbance of various FSP-made wide and mid-bandgap photocatalysts, as well as some of the more common organic substrates. Wide absorbance organic dyes such as methylene blue and rhodamine B are difficult to avoid photosensitisation. To complicate things, it is not uncommon to observe the shifting of the absorbance profile during the course of dyes degradation. Non-dye substrates are in general less problematic as they rarely have overlap absorbance with the assessed photocatalysts. As precautions, even when assessing the photocatalytic degradation of aromatic compounds (absorbance < 300 nm), cut off filters below their absorbance can be coupled to the light source to avoid photosensitisation.

## 3. *In situ* Flame-doping of Photocatalysts—A Small Addition Makes Big Changes

Doping of metal oxide photocatalysts can be conveniently carried out in FSP by co-dissolving foreign metal precursors in the overall precursor formulation. Because of the well-controlled stoichiometry of both the parent metal elements and that of dopants, this allows precise engineering of the dopant concentrations during synthesis, almost unparalleled by other doping techniques [33]. However, the challenge has been to achieve the desirable specific physicochemical states of the dopants. They include the control of substitutional or interstitial doping, whether the dopants are surface concentrated or distributed homogeneously as solid solutions, or whether the dopants maintain the desirable oxidation states and coordination number,* etc.* More specific questions pertaining to photocatalysts design include, how can one obtain a surface-enriched doping of Rh^3+^ in SrTiO_3_, instead of being homogeneously distributed? Can we obtain more V^4+^ doping instead of V^5+^ on TiO_2_ surface, or can we obtain exclusive interstitial instead of substitutional Ti^4+^ doping in Fe_2_O_3_? Despite the importance of these outcomes that are in turn vital to the successful design of the doped photocatalysts, many of these challenges in FSP synthesis remain underexplored.

**Figure 4:**
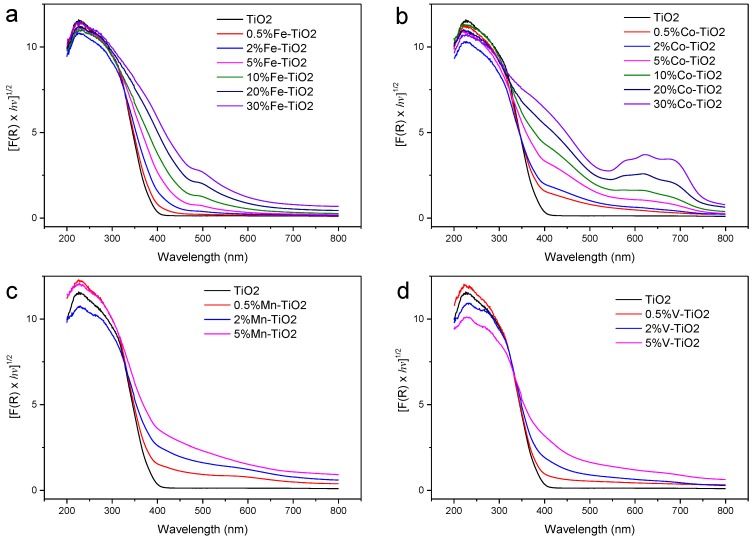
Tauc plot of pristine and metal-doped TiO_2_ at various loadings (expressed in atomic percentage with respect to Ti) prepared in a one-step FSP. The metal dopants include (**a**) Fe; (**b**) Co; (**c**) Mn and (**d**) V.

In search of visible light-activated photocatalysts, we reported the FSP synthesis of Fe^3+^-doped TiO_2_ photocatalyst that was active in the mineralisation of oxalic acid [65]. Doping Fe^3+^ in TiO_2_ created sub-bandgap energy levels from which electrons can exist and be photoexcited to conduction band with lower energy photons,* i.e.*, visible light (Figure 4a) [66]. Because of the extremely high synthesis temperature and rapid quenching of the aerosol flame (from radiation heat loss and air entrainment), homogeneous doping as high as Fe/Ti = 0.05 can be achieved from FSP synthesis, which is 2.5 times higher than traditionally prepared by wet techniques. For this, we found that Fe-doped TiO_2_ (Fe/Ti = 0.02) exhibited optimal visible light activity. However, the mechanism of oxalic acid mineralisation over Fe-doped TiO_2_ was not a simple photohole oxidation. Such insight was only obtained when following the stability of the dopant during the course of the reaction. An intriguing homogeneous-heterogeneous reaction actually took place with initial complexation-leaching between surface Fe(III) and adsorbed oxalic acid to form Fe(III)-oxalate, which decomposes under visible light [65]. This results in free Fe(II) that then readsorbs on the Fe–TiO_2_ surface, and reoxidized by photoholes to regenerate Fe(III) [65]. The unique leaching and readsorption allows recovery of all Fe dopants to be reused effectively.

As with any metal-doped photocatalysts, it is essential to investigate the stability of the dopants to gain real insights into its degradation mechanism. One should always assume that dopants are unstable, unless proven otherwise. There are only some isolated cases, such as Fe-doped ZnO [67], where the dopants actually help increase aqueous stability of the photocatalysts. Here, the stability was thought to originate from the formation of zinc ferrite-like phase [68].

By extension of the one-step FSP synthesis of Fe-doped TiO_2_, similar attempts were made with V, Co and Mn dopants. However, these dopants only exhibit partial (shoulder like) extension of the TiO_2_ intrinsic absorption edge, which is likely due to inhomogeneity of the metal dopants (Figure 4). Vanadium, for example, tends to concentrate on the TiO_2_ surface during flame synthesis [69]. Tian* et al.* [70] reported enhancement in the degradation of methylene blue dye over FSP-made V-doped TiO_2_compared to bare TiO_2_ under both UV (optimal 0.5% V–TiO_2_) and visible light irradiation (optimal 1% V–TiO_2_). Evidence of V^4+^ substitutional doping was suggested based on EPR spectroscopy, alongside surface layer V^5+^ which was otherwise electroparamagnetically invisible [70,71]. In general, phase segregation any dopants ought to occur when the solid-state solubility limit is exceeded. Figure 4b shows the segregation of cobalt oxide at >5 at % loading as evident from the 550–750 nm band.

Besides modifying the band structure of the parent oxide photocatalyst, doping has pronounced effects on the hydroxyl group density. By using FSP-made photocatalysts, Jiang* et al.* [72] showed the increase in photocatalytic oxidation of acetaldehyde with decreasing terminal Ti–OH group achieved by doping. The hydroxyl group density follows the order of Cu-doped TiO_2_ > FSP TiO_2_ > P25 > F-doped TiO_2_. Unlike aqueous phase photocatalysis, less hydroxyl groups allows more efficient direct charge transfer, which is the dominant mechanism in the gas phase photocatalytic oxidation.

## 4. Flame-Made Mixed Metal Oxide Photocatalysts—Trying out Complexities

To date, the variety of FSP-synthesised mixed metal oxide photocatalysts has been limited despite its huge potential for the discovery of new materials through bandgap engineering, a strategy widely adopted in the design of water splitting photocatalysts [73]. By comparisons, the FSP synthesis of mixed metal oxides in other applications has made more significant progress in terms of the number of successful high-performance materials synthesised: For examples, thermal catalysis (Ce*_x_*Zr_1−*x*_O_2_ three-way catalysts [40], CuO-CeO_2_ PrO*_x_* [23,24] and *de*NO*_x_* catalysts [25], V_2_O_5_/TiO_2_ oxidation catalyst) [69], Li-ion batteries (LiMn_2_O_4_, Li_4_Ti_5_O_12_, LiFe_5_PO_8_) [37,74], gas sensors (In_4_Sn_3_O_12_) [75] and biomaterials (hydroxyapatite [76], dental fillers [77], bioglass [78]). Like the general FSP syntheses of photocatalyst nanoparticles, the challenge lies in the synthesis of highly crystallined and defects-free nanoparticles, except that the challenge is drastically augmented with increase in the number of metal elements. As such, only a couple of cases of decent mixed oxide photocatalysts have been reported thus far.

BiVO_4_ is an active visible light-driven photocatalyst with highly oxidising valence band edge. Strobel *et* *al.* [79] reported the FSP synthesis of this vivid yellow pigment with a bandgap of ~2.5 eV. The synthesis of BiVO_4_ by FSP is an interesting one in that the BiVO_4_ aerosol leaving the flame is actually in an amorphous state. However, the later exposure to high temperature heat directly above the flame during filter-collection (*T* > 310 °C) resulted in the crystallisation of BiVO_4_,* i.e.*, on the filter [79]. In as-collected form, the BiVO_4_ shows decent activity in the photobleaching of methylene blue [80]. By controlling precisely the filter exposure temperature, one is able to tune the polymorphic phase of the BiVO_4_ crystal, which in turn reflects its photocatalytic activity. At relatively low temperature but above crystallisation temperature, photoactive scheelite-monoclinic phase can be obtained up to 430 °C, above which the less active scheelite-tetragonal phase will dominate [43]. To further enhance the photocatalytic activity of the BiVO_4_, the large presence of oxygen vacancies and other defects ought to be overcome. Post-treatment with aqueous acid-dissolved bismuth nitrate and vanadia was found to “heal” these recombination centres and significantly improved the photocatalytic oxygen evolution under visible light by 5-fold [43].

Akurati* et al.* [81] introduced the WO_3_/TiO_2_ composite photocatalyst to tune the surface Brønsted and Lewis acidity of the TiO_2_. This is a common strategy in thermal catalysis, which when applied to photocatalysis, has the additional role of improved charge separation across the heterojunctions of TiO_2_ and WO_3_, as shown for the enhanced degradation of methylene blue [81]. In this regards, the transfer of accumulated photoelectrons on W^5+^ would require the deposition of Pt or Cu^2+^ co-catalyst for the multiple electron reduction of oxygen (as mentioned earlier) [61].

As we see it, there are two major hurdles that need to be overcome to pave the way for more successful synthesis of mixed oxide photocatalysts by FSP: (i) The first being the quality of the resultant mixed oxide crystals. It appears that for composite photocatalysts, especially those of complex oxides, much longer residence time at high flame temperature is often required to achieve adequate crystallisation and minimising of charge trap defects. This will however compromise on the specific surface area (or crystallite size). Although the quartz-tube enclosed can prolong the required residence time, we found in the (perhaps isolated) case of BiVO_4_ where the accompanied high temperature flame resulted in the unfavourable high temperature zircon phase and sublimation of Bi, resulting in even more defects; (ii) The second criterion is the limitation of precursors. Although we did mention earlier that one of the main advantages of FSP lies in its innate ability to utilise liquid precursors, more exotic metallorganic precursors such as those containing Ga, Ta, Sr, Sb, La and Pr, which are commonly used elements in the design of mixed oxide photocatalysts, are either still unavailable or too expensive. In this respect, the upgrading of these metal precursors from low cost metal nitrates, acetates or even oxides to suitable metallorganics remains a major bottleneck.

## 5. Flame Deposition of Noble Metals on Oxide Photocatalysts

The deposition of noble metals is a classical technique for enhancing charge separation and the photocatalytic reaction rates. At the metal-semiconductor interface, Schottky barrier is formed due to the difference between Fermi energy level and the conduction band edge of the semiconductor photocatalyst. This prevents trapped photoelectrons from back flowing to the photocatalyst conduction band, and hence better charge separation can be achieved. The noble metal/photocatalyst structures explored by FSP include Pt/TiO_2_ [47], Ag/TiO_2_ [27], Au/TiO_2_ [82] and Ag/ZnO [57]. The segregation of the noble metals as nanosize deposits during FSP relies on the fact that (i) the noble metal has relatively low boiling/sublimation point of the metal compared to oxide photocatalyst, so that the former precipitates at a later stage in the flame after the precipitation of the oxide photocatalyst support, and/or (ii) low solubility of the two phases which otherwise enhances bulk doping rather than surface deposition. Recalling the effects of metal precursors that determine if the FSP-made particles are formed by the gas-to-particle or droplet-to-particle route, the same principle applies to the deposition of noble metals. Typically, the use of noble metal acetylacetonates and carboxylates ensure the formation of homogeneous deposits (of 2–5 nm) [27] through the gas-to-particle precipitation, while noble metal nitrates result in inhomogeneous deposits (2–50 nm) by the droplet-to-particle route [57].

All the photocatalysis cases reported so far on the deposition of noble metal by FSP has resulted in the enhancement of photocatalytic activities (that is at optimal concentration), compared to the corresponding bare oxides. Typically, an optimum concentration of exists, beyond which the metal deposits become recombination centres and at even higher concentration may result in the shielding of light absorption. This is true irrespective of the organic test substrates, be it saccharides [47], carboxylic acids, alcohols, aromatics [83] or dyes [57]. Perhaps quite differently from the typical photocatalytic assessments, Chiarello* et al.* [84,85] demonstrated the photocatalytic reforming of gaseous methanol over FSP-made Pt/TiO_2_ to form carbon monoxide and hydrogen. The FSP-prepared Pt/TiO_2_ showed drastically higher photocatalytic activity than that prepared by wet technique as a result of higher noble metal dispersion.

Strictly speaking, the oxidation states of the as-prepared metal deposits are in oxide forms, at least shown for Ag_2_O [27] and PtO [47,83] on TiO_2_. Because these noble metals are electrons traps, they can be easily reduced under excitation of the photocatalyst support. An intricate phenomenon that has often been overlooked is the dynamic of oxidation states of the noble metal deposits, which besides the Schottky electron trapping, is deeply correlated with the mechanistic pathway of the photocatalytic reactions. For instance, the photocatalytic oxidation of formic acid results in the formation of highly reducing COO· radical [84], which readily reduces PtO to metallic Pt [83]. The photocatalytic degradation of aromatic-containing compounds involves a null redox cycle between hydroquinone and benzoquinone [4], where the reduction of benzoquinone to hydroquinone results in the oxidation of Pt deposits and vice versa for the cyclic reoxidation to benzoquinone. As such the initial Pt(II) state may, at the end of reaction, exist in mixed oxidation states Pt(0, II and IV), the composition of which reflects the overall reaction path [83]. Additionally, the co-catalytic effects of noble metal deposits may be beneficial and contributing to the overall photocatalytic reaction, and hence shall not be completely ignored.

**Figure 5:**
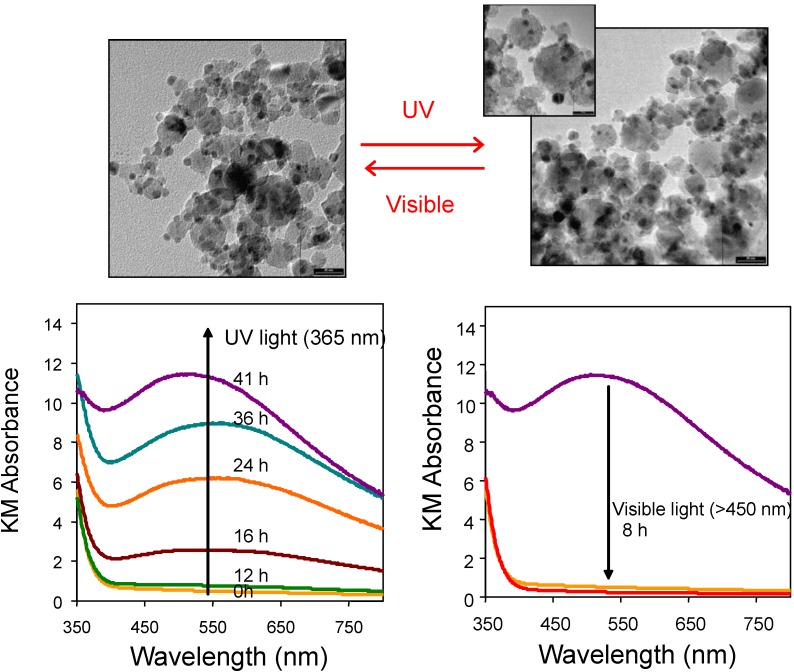
Transmission electron micrographs of FSP-made Ag/TiO_2_ samples after UV and visible light irradiation. Under UV irradiation, Ag(I) oxide deposits are photoreduced and result in the increase in surface plasmon resonance as characteristics of metallic Ag. Irradiation by visible light resulted in the injection of excited electrons from metallic Ag to the conduction band of TiO_2_, and in the process oxidising it back to Ag(I) oxide. This resulted in the loss of surface plasmon resonance effects. Adapted with permission from [26].

Since metal deposits can be reduced to metallic state upon photoexcitation of the photocatalysts, this gives rise to the surface plasmon resonance, as evident in the visible wavelengths for metals such as Ag and Au. As shown in Figure 5, Ag(I) and Ag(0) can inter-switch reversibly by irradiating Ag/TiO_2_ photocatalyst with UV (Ag(0) to Ag(I)) and visible light (Ag(I) to Ag(0)) [27]. Perhaps interesting to mention, the surface plasmon absorption by metal deposits can generate a localised electric field that may enhance charge separation on the photocatalyst and the resultant photocatalytic activities [86]. However, it is to date difficult to decouple, at least from the photocatalytic asessments alone, the combination of effects from Schottky barrier, co-catalyst and surface plasmon resonance.

## 6. Concluding Remarks

The integration of FSP as a mainstream technique for photocatalysts synthesis is still in its dawn. Whilst some design strategies established for the synthesis of thermal catalysts may be transferable to the designing of photocatalysts, the priorities of these design criteria can be quite different. Experience gained from the synthesis of simple metal oxide photocatalysts, has identified perhaps the most fundamental challenge that ought to be overcome: photocharge trap defects arising from the extremely short flame residence time. Overcoming this very challenge is even more daunting for mixed oxide photocatalysts, but one that holds unprecedented potential for novel photocatalysts design. The same challenge applies to the general FSP synthesis of other functional materials involving charge transport (solar cells), electrons spin (superconductor, magnetic and spintronic applications), and energy transfer (phosphors), for which efficiencies are highly dependent on the quality of the nanocrystals. An area that FSP may prevail over the more established wet synthesis of photocatalysts, in addition to the readily scalable process to industrial mass, is the ability to yield metastable photocatalysts. These metastable materials are obtained in part because of the short flame residence time at extreme conditions. If photocatalysts can be designed and at the same time overcoming the inherent charge trap defects and recombination centres, this can potentially result in a new class of efficient photocatalysts that is unique to flame synthesis.
